# Regional and interhemispheric differences of neuronal representations in dentate gyrus and CA3 inferred from expression of zif268

**DOI:** 10.1038/s41598-023-45304-y

**Published:** 2023-10-27

**Authors:** Lars-Patrick Schmill, Katharina Bohle, Niels Röhrdanz, Thomas Schiffelholz, Kira Balueva, Peer Wulff

**Affiliations:** 1 Institute of Physiology, Christian-Albrechts-University Kiel, Kiel, Germany; 2 Center of Integrative Psychiatry, University Medical Center Schleswig-Holstein, Kiel, Germany; 3 Present Address: Clinic for Radiology and Neuroradiology, UKSH, Kiel, Germany; 4 Present Address: Clinic for Orthopaedic and Trauma and Reconstructive Surgery, Klinikum Frankfurt Höchst GmbH, Frankfurt am Main, Germany

**Keywords:** Fluorescence in situ hybridization, Neurophysiology, Cellular neuroscience, Hippocampus, Spatial memory, Neural circuits

## Abstract

The hippocampal formation is one of the best studied brain regions for spatial and mnemonic representations. These representations have been reported to differ in their properties for individual hippocampal subregions. One approach that allows the detection of neuronal representations is immediate early gene imaging, which relies on the visualization of genomic responses of activated neuronal populations, so called engrams. This method permits the within-animal comparison of neuronal representations across different subregions. In this work, we have used compartmental analysis of temporal activity by fluorescence in-situ hybridisation (catFISH) of the immediate early gene zif268/erg1 to compare neuronal representations between subdivisions of the dentate gyrus and CA3 upon exploration of different contexts. Our findings give an account of subregion-specific ensemble sizes. We confirm previous results regarding disambiguation abilities in dentate gyrus and CA3 but in addition report novel findings: Although ensemble sizes in the lower blade of the dentate gyrus are significantly smaller than in the upper blade both blades are responsive to environmental change. Beyond this, we show significant differences in the representation of familiar and novel environments along the longitudinal axis of dorsal CA3 and most interestingly between CA3 regions of both hemispheres.

## Introduction

During learning individual hippocampal regions differently contribute to memory formation^1^. Information regarding the nature of these contributions can be derived from the region-specific analysis of neuronal representations^2–4^. One well studied example for differential region-specific functions within the hippocampus concerns its ability to separately encode similar but distinct memories and, at the same time, support the retrieval of memories from partial cues. Based on network architecture, computational models have predicted that these complementary tasks are separated along the sub-circuits of the transverse axis. Specifically, it has been hypothesized that the former task may be mediated by the dentate gyrus (DG) in a process termed pattern separation and the latter by CA3 in a process termed pattern completion^5–9^. Here pattern separation describes the ability to reduce the overlap of neuronal representations from entorhinal cortex to DG by means of recoding information onto a larger but sparsely active granule cell population. Pattern completion on the other hand describes the ability to generate a full neuronal representation from reduced or partial input and is thought to be accomplished through the dense recurrent collateral system of CA3. These models are supported by place cell recordings, which have shown that indeed DG and proximal CA3 show strong population remapping in response to changes in environment, whereas neuronal ensemble activity in distal CA3 showed reduced remapping with mild environmental change^10–15^.

In agreement with differences in connectivity, gene expression patterns and neuronal morphology^16–20^ it has been proposed that functional specializations along the transverse axis of the hippocampus may not be homogeneously implemented along the rostro-caudal axis or between hemispheres^21–25^. Here we set out to investigate how neuronal representations evoked by brief exposures to new or familiar environments would differ along anatomical axes of DG and CA3 and between hemispheres.

To do so we have used a method termed immediate early gene (IEG) imaging, which is well suited to investigate representations on the population level across larger volumes. It relies on the detection of genomic responses of contextually activated neuronal populations, which in turn correspond to contextual memory engrams in the hippocampus^2, 3, 26^. IEGs are rapidly transcribed following strong synaptic stimulation as encountered during the induction of synaptic plasticity or during learning and their protein products are generally thought to directly or indirectly modify cellular function in response to these stimuli^27^. Prominent examples of IEGs, which have been used for the detection of behaviour related hippocampal representations are Arc and zif268, which both play crucial roles in synaptic plasticity and spatial learning^27–29^. These IEGs can also be used to disambiguate mnemonic representations of two sequential contextual exposures, a technique termed compartment analysis of temporal activity by fluorescent in-situ hybridization (catFISH), which is based on the time-dependent transport of IEG-mRNA from the nucleus to the cytoplasm^2^. The dynamics of this transport have been established for Arc as well as zif268 mRNA in the hippocampal CA1 region: 5 min after induction mRNA is exclusively detected in nuclei (maximum of positive nuclei), then the mRNA starts to shuttle into the cytosol. After 30 min both mRNAs are only detected in the cytosol (maximum of cytosolic signals). These dynamics determine the catFISH protocol: 1st exposure 5 min, interval 20 to 30 min, second exposure 5 min. This protocol has since been used successfully to discriminate sequential representations in different hippocampal subregions^2, 30–37^ and has provided support for a role in pattern separation and pattern completion in DG and CA3, respectively^30, 31, 36, 37^. However, neuronal representations as defined by IEG expression in DG and CA3 have not been compared directly within the same experiment or along the longitudinal axis of dorsal hippocampus or between hemispheres.

Since Arc, which is the most widely used IEG in catFISH experiments shows transcriptional dynamics in the DG, which deviate from other hippocampal regions^31, 38^ we have turned to zif268^28, 29, 36, 37^ to simultaneously investigate neuronal representations of two sequential environments in different subregions of DG and CA3. We show that sequential representations have more overlap in distal CA3 than DG for exposures to unaltered environments but not for exposures to distinct environments. In addition, we show that despite differences in the size of activated ensembles both the suprapyramidal (spDG) and the infrapyramidal blade of the DG (ipDG) are responsive to environmental change. Finally, we describe differences in neuronal representation along the longitudinal axis of the dorsal hippocampus and remarkably between brain hemispheres.

## Results

### Compartment specific visualization of zif268 transcripts permits time-dependent segregation of activated ensembles in DG and CA3

**Figure 1:**
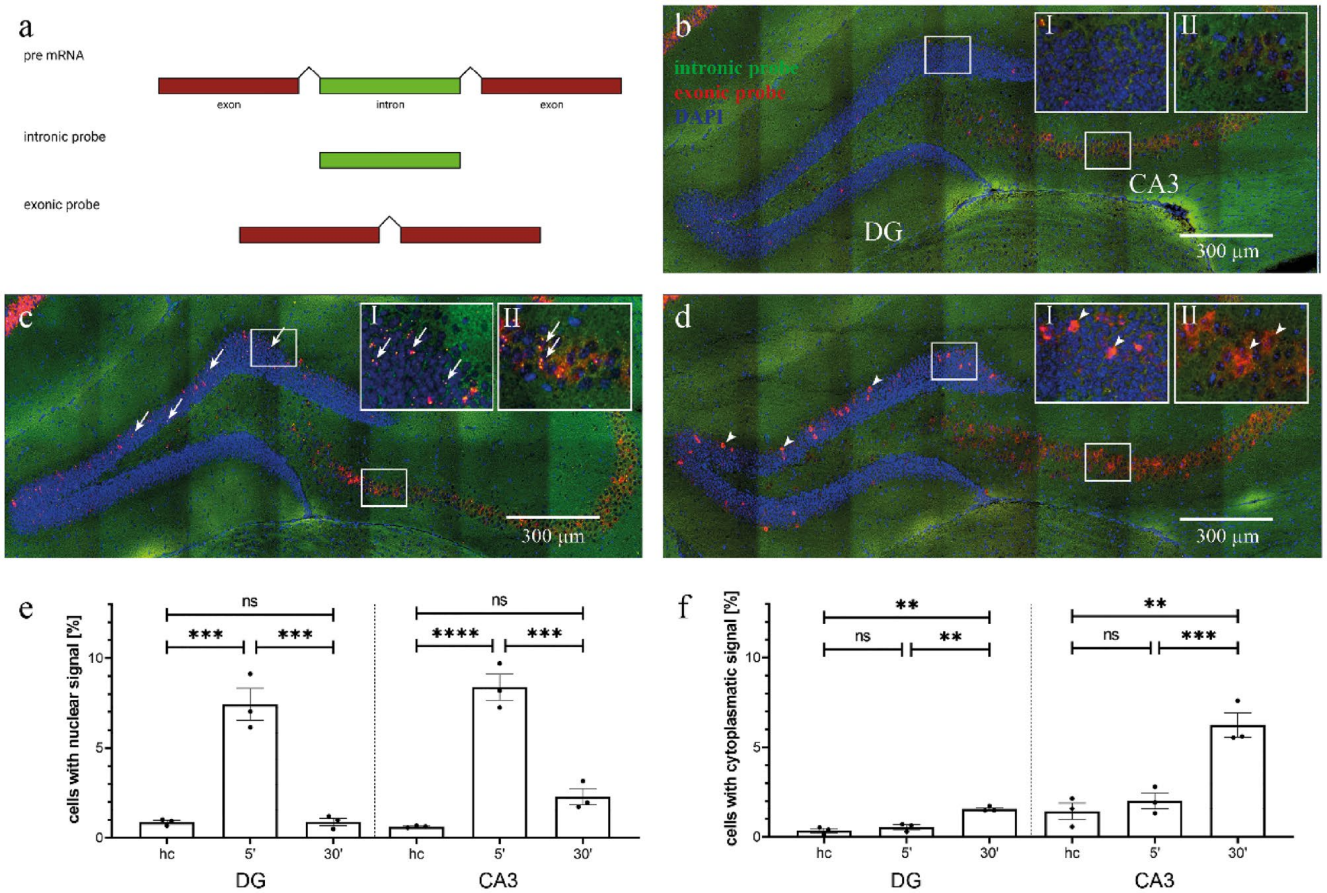
Double fluorescent in situ hybridization for zif268 reliably distinguishes sequential exposures separated by 30 min. (**a**) Intron containing pre-mRNA is localized in the nucleus. After removal of introns mRNA is shuttled to the cytosol. Probes are directed against intronic or exonic sequences, respectively. (**b–d**) Example images of coronal hippocampal sections from (**b**) home cage, (**c**) 5’ and (**d**) 30’ hybridized with exonic (red) and intronic (green) probes and stained for nuclei with DAPI (blue). Insets show magnifications of boxed areas. Arrows mark nuclear signals, arrowheads indicate cytoplasmatic signals. Scale bars are 300 µm. (**e**) Nuclear signals were strongly increased 5 min after exposure to a new environment in both DG and CA3 but returned to home cage (hc) levels 30 min after the exposure. (**f**) Cytoplasmatic signals were at hc levels 5 min after exposure to a new environment but significantly increased 30 min after the exposure. Stars indicate the level of significance of the results of Bonferroni’s multiple comparisons test as post-hoc test for two-way ANOVA. ns = not significant; **p < 0.005; ***p < 0.0005; ****p < 0.0001.

To easily distinguish cytoplasmatic and nuclear zif268 in situ signals we generated 2 separate probes: one that detects mRNA (directed against exonic sequence, visualized by red fluorescence) and one that detects only pre-mRNA (directed against intronic sequence, visualized by green fluorescence), respectively (Fig. 1a)^39^.

To gauge how reliable nuclear and cytoplasmatic zif268 signals would identify temporally separated neuronal representations using established catFISH protocols, we tested our probes on 3 groups of mice: 1.) hc, composed of naïve mice, which were taken directly from the home cage; 2.) 5’, which had visited a new context for 5 min immediately before the brain was taken and 3.) 30’, which were taken from the home cage 25 min after they had explored a new context for 5 min (Fig. 1b,c,d). As expected for a time-dependent shift between compartments we found the percentage of neurons with nuclear zif268 signals in DG and CA3 to be significantly elevated immediately after exploration of a novel environment but back to base line (home cage) 25 min after exploration (DG: F_2, 6_ = 51.23, *p* = 0.0002; CA3: F_2, 6_ = 70.09, *p* < 0.0001; one-way ANOVA; DG: hc vs 5’: *p* = 0.0004, hc vs 30’: *p* > 0.9999, 5’ vs 30’: *p* = 0.0004; CA3: hc vs 5’: *p* < 0.0001, hc vs 30’: *p* = 0.1561, 5’ vs 30’: *p* = 0.0004; Bonferroni multiple comparisons test; Fig. 1e). In contrast, the percentage of neurons with cytoplasmatic zif268 signals in DG and CA3 were similar in the hc and 5’ groups but significantly increased in the 30’ group (DG: F_2, 6_ = 32.81, *p* = 0.0006; CA3: F_2, 6_ = 24.02, *p* = 0.0014; one-way ANOVA; DG: hc vs 5’: *p* = 0.8073, hc vs 30’: *p* = 0.0008, 5’ vs 30’: *p* = 0.0022; CA3: hc vs 5’: *p* > 0.9999, hc vs 30’: *p* = 0.0021, 5’ vs 30’: *p* = 0.0042; Bonferroni multiple comparisons test; Fig. 1f). These data are in line with the transcriptional dynamics reported for CA1 and corroborate previous catFISH protocols used in CA1, CA3 and DG^2, 30, 34–37^. They indicate that compartment-specific detection of zif268 mRNA allows the distinction of neuronal populations activated at time points that are about 30 min apart.

### Sequential exposure to the same or different environments activates similar percentages of neurons

**Figure 2:**
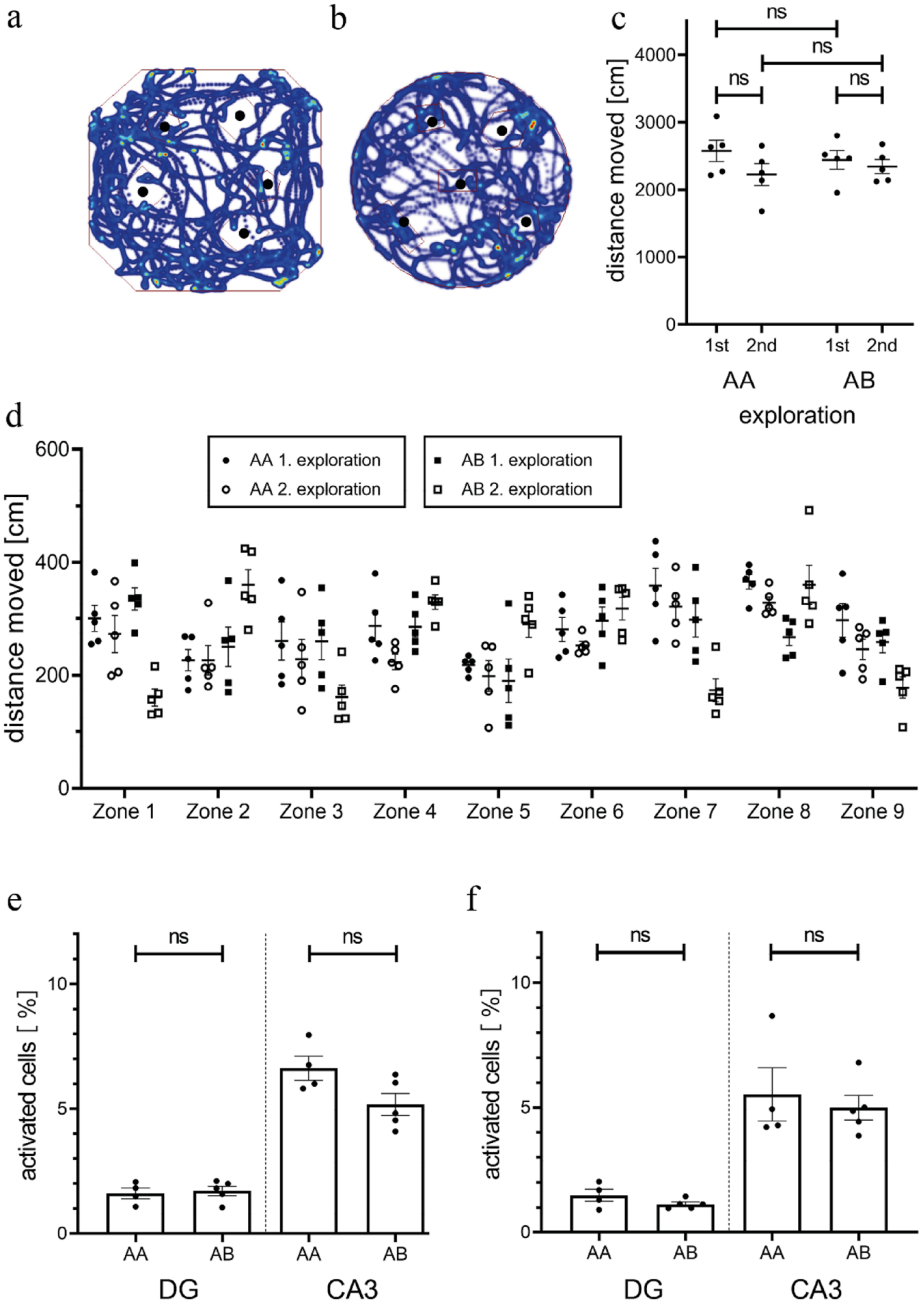
Both environments are explored equally and activate a similar percentage of neurons in DG and CA3. (**a**) and (**b**) show examples of movement tracks in context A (**a**) and context B (**b**). Black dots indicate the position of objects. (**c**) We found no differences in the total distance moved between the different exposures of the AA and AB groups. (**d**) All mice showed similar exploration of the environments during individual exposures as indicated by the distance moved in individual zones. (**e, f**) Neither the percentage of neurons with nuclear (**e**) nor with cytoplasmic (**f**) zif268 catFISH signals in DG and CA3 was significantly different between the individual exposures of the AA or AB groups. ns = not significant.

**Figure 3:**
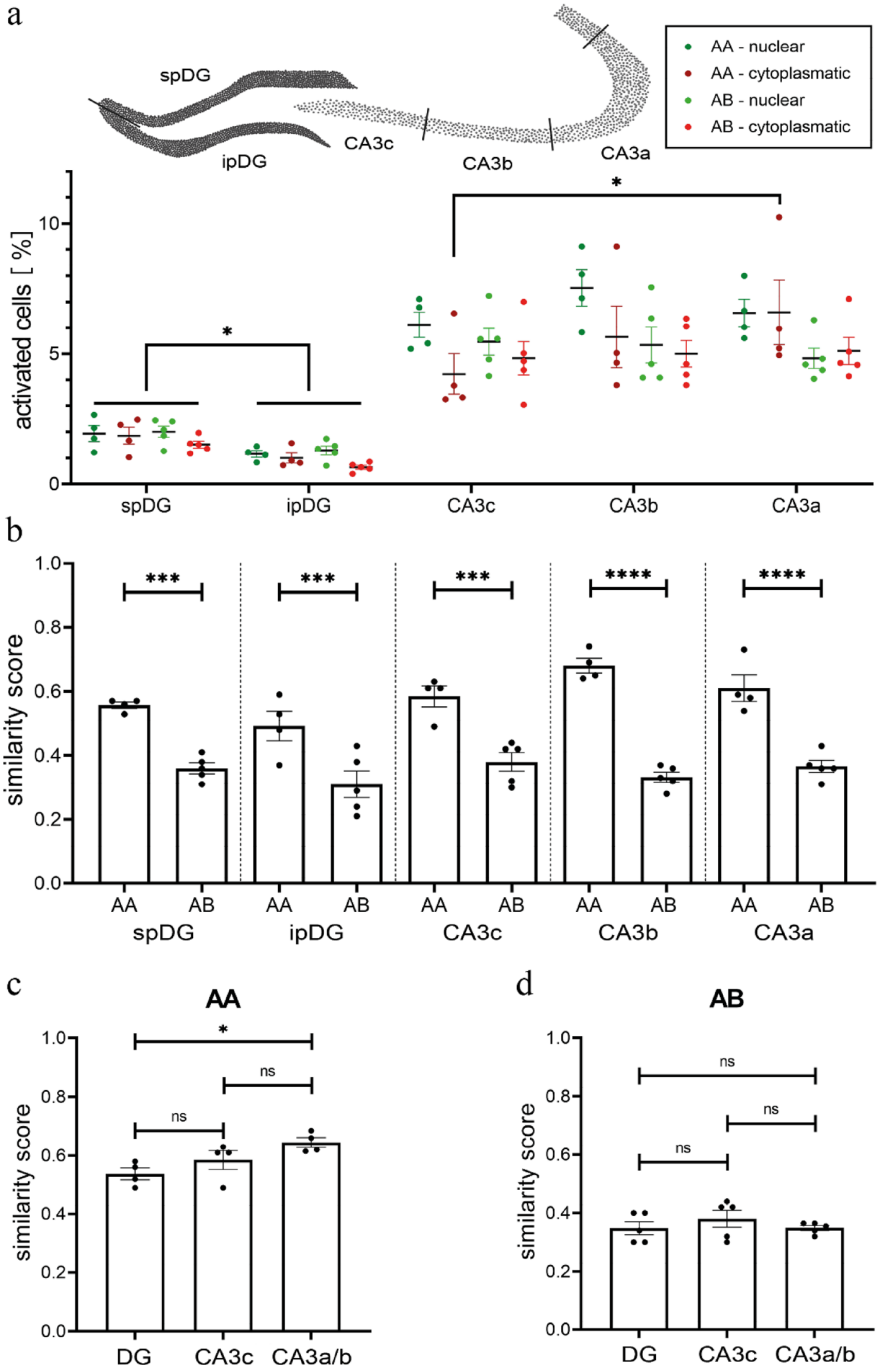
Ensemble sizes and similarity scores in sub-regions of DG and CA3. (**a**) Top: Schematic illustrating the division of DG and CA3 into subregions. Bottom: A significantly higher percentage of neurons shows nuclear or cytoplasmic signals in the spDG than in the ipDG in both the AA and the AB group. Within CA3, percentages of activated neurons do not show consistent differences. Only in group AA a higher number of cytoplasmic signals was detected in CA3a than in CA3c. (**b**) For all subregions of the DG and CA3, the similarity score was significantly higher for repeated exploration of context A (AA) than for exploration of two different environments (AB). (**c**) The similarity score in the AA group is significantly higher in CA3a/b than in the DG. (**d**) For group AB, no differences in similarity score were obvious between the subregions of CA3 and the DG or within CA3. ns = not significant; *p < 0.05; ***p < 0.0005; ****p < 0.0001.

To investigate the features of sequential neuronal representations for two environments we exposed two new cohorts of mice either twice to the same environment A (AA group) or first to environment A and then to environment B, which was located in the same room but differed from A regarding the foreground cues (AB group; see methods, Vazdarjanova and Guzowski^30^). Exposures lasted 5 min with an interval of 25 min. All mice showed similar exploration of the two environments as indicated by both the total distance moved during each exposure (groups: F_1, 16_ = 0.0027, *p* = 0.9589; exposures: F_1, 16_ = 2.456, *p* = 0.1366; two-way ANOVA) as well as by the coverage of space within each arena (Fig. 2a–d, see methods). Analysis of the percentage of neurons with nuclear or cytoplasmatic zif268 signals in the DG or CA3 showed no difference between the AA and AB groups (Fig. 2e,f; DG: groups: F_1,14_ = 0.4984, *p* = 0.4918; signal type: F_1,14_ = 3.385, *p* = 0.0871; CA3: groups: F_1,14_ = 2.462, *p* = 0.1389; signal type: F_1,14_ = 1.013, *p* = 0.3313; two-way ANOVA), suggesting that all exposures activated similar numbers of neurons. In both groups the percentage of neurons with either nuclear or cytoplasmatic signals was about three-fold higher in CA3 than in DG (Fig. 2e,f; nuclear: regions F_1,14_ = 140.4, *p* < 0.0001; groups F_1,14_ = 3.632, *p* = 0.0774; two-way ANOVA; DG vs CA3: AA *p* < 0.0001, AB *p* < 0.0001; Bonferroni’s multiple comparisons test; cytoplasmic: regions F_1,14_ = 50.38, *p* < 0.0001; groups F_1,14_ = 0.6375, *p* = 0.4380; two-way ANOVA; DG vs CA3: AA *p* = 0.0005, AB *p* = 0.0003; Bonferroni’s multiple comparisons test), in line with previous studies^2, 31, 37^. To assess whether the size of neuronal representations also differed between individual subregions, we divided the DG into suprapyramidal and infrapyramidal blade and CA3 into zones a, b, c (see Fig. 3a; Nó^40^). Indeed, we found the percentage of activated neurons to be significantly higher in spDG than in ipDG (Fig. 3a; nuclear: blades F_1, 14_ = 12.55, *p* = 0.0032; groups F_1, 14_ = 0.2314, *p* = 0.6380; two-way ANOVA; cytoplasmatic: blades F_1, 14_ = 21.23, *p* = 0.0004; groups F_1, 14_ = 3.506, *p* = 0.0822; two-way ANOVA), see also Satvat et al.^41^. However, between the subregions of CA3 the percentage of activated neurons was similar with the exception of one significant difference between CA3a and CA3c for cytoplasmatic signals in the AA group (Fig. 3a; nuclear CA3 a, b, c: AA: F_1.4, 4.2_ = 4.336, *p* = 0.0986, AB: F_1.5, 6.0_ = 0.9631, *p* = 0.9631; cytoplasmatic CA3 a, b, c: AA: F_1.4, 4.3_ = 19.41, *p* = 0.0046, AB: F_1.5, 6.0_ = 0.1810, *p* = 0.7800; repeated measures one-way ANOVA with Greenhouse–Geisser correction; CA3a vs CA3c for cytoplasmic signals in the AA group: *p* = 0.0025; Bonferroni’s multiple comparisons test).

### DG and CA3 show similar distinction between two different environments at the population level

To investigate whether neuronal representations in DG and CA3 as well as their subregions differed regarding their ability to disambiguate environment A from environment B, we analyzed the degree of overlap between the two sequential representations in the AA and AB groups. As a measure of overlap we have used the similarity score, which calculates the fraction of activated neurons which participate in the representation of both environments^30^. As expected, the similarity score was significantly higher in the AA than in the AB group for both DG and CA3 (Similarity score: F_1, 14_ = 122.8, *p* < 0.0001; two-way ANOVA; AA vs AB: DG *p* < 0.0001, CA3 *p* < 0.0001; Bonferroni’s multiple comparisons test) and all subregions analyzed (Similarity score: F_1, 35_ = 158.6, *p* < 0.0001; two-way ANOVA; AA vs AB: spDG *p* = 0.0002, ipDG *p* = 0.0005, CA3c *p* = 0.0001, CA3b *p* < 0.0001, CA3a *p* < 0.0001; Bonferroni’s multiple comparisons test; Fig. 3b). Thus, the more similar the two environments were, the more likely it was that neurons that participated in the first representation were part also of the second and vice versa. Previous work has suggested that the DG and possibly CA3c may participate in pattern separation, whereas CA3a and CA3b maybe involved in pattern completion or rather pattern convergence^42, 43^. Indeed, we found a higher degree of overlap of neuronal representations for the AA group in CA3a/b than in DG (Similarity score: H_2_ = 6.865, *p* = 0.0197; Kruskal–Wallis test; CA3a/b vs DG *p* = 0.0273; Dunn’s multiple comparisons test) whereas CA3c was not significantly different from DG (Similarity score: CA3c vs DG *p* = 0.8372, CA3c vs CA3a/b *p* = 0.3816; Dunn’s multiple comparisons test; Fig. 3c). The similarity score for neuronal representation of two more dissimilar environments (AB group) were not significantly different between CA3a/b and DG (Similarity score: F_2, 12_ = 0.7005, *p* = 0.5155; one-way ANOVA; CA3a/b vs DG: *p* > 0.9999; Bonferroni’s multiple comparisons test; Fig. 3d). Similar differences along the transversal axis of CA3 were detected using Arc/Arg3.1 catFISH^37^.

In summary we found that the overlap between activated ensembles was significantly higher in the AA than the AB group for all subregions and that CA3a/b showed higher overlap than its input region the DG in the AA but not the AB group.

### Septo-temporal and inter-hemispheric differences in neuronal representations

**Figure 4:**
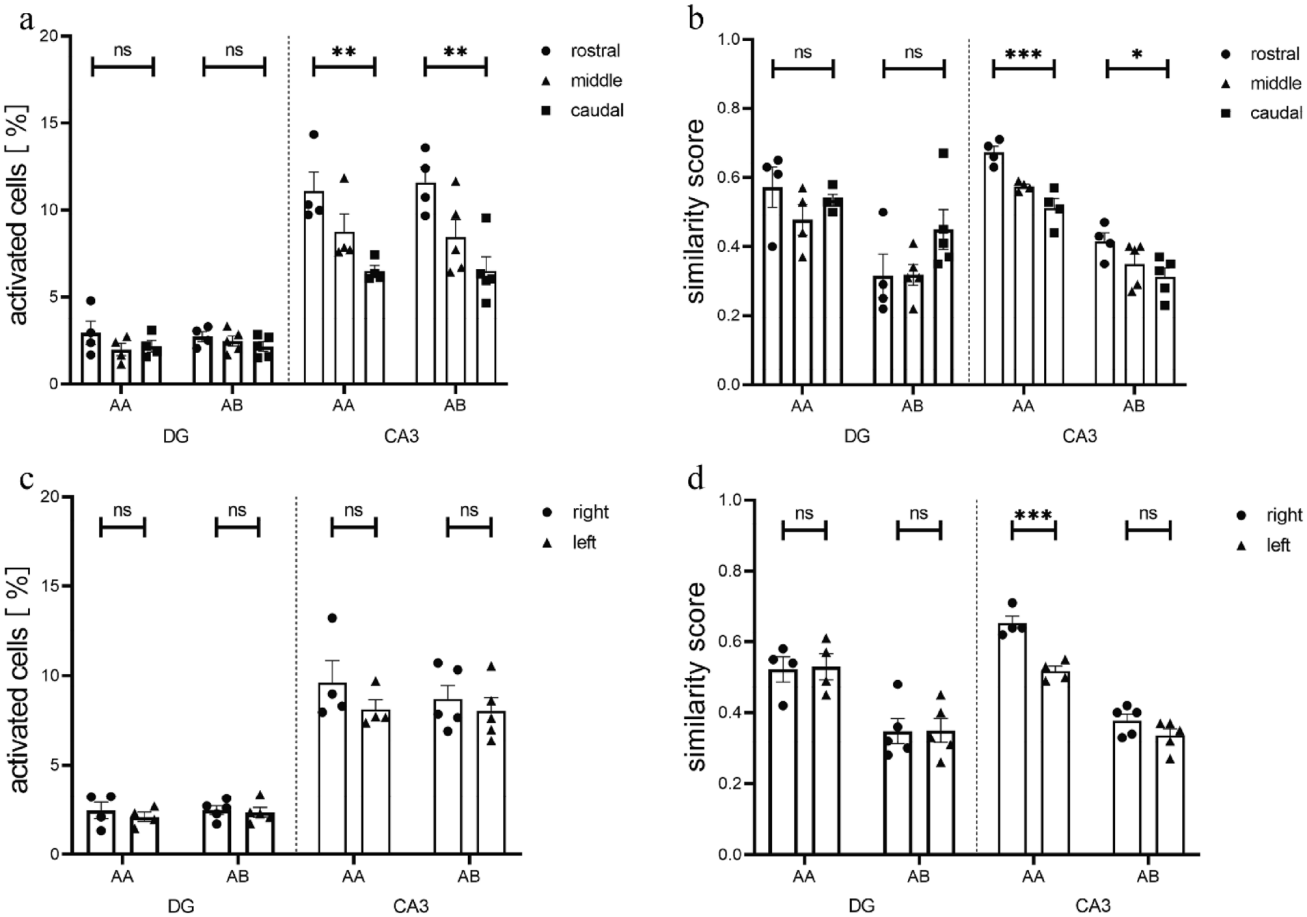
Ensemble sizes and similarity scores differ along the rostro-caudal axis and between hemispheres. (**a**) Along the rostro-caudal axis of the dorsal hippocampus the percentage of activated neurons does not change in the DG but is significantly larger in rostral than caudal parts of CA3. (**b**) The similarity scores for both groups were significantly higher in rostral than caudal parts of CA3. (**c**) The percentage of activated neurons was not significantly different between hemispheres in either the AA or the AB group. (**d**) The AA group showed significantly higher similarity scores in CA3 of the right than the left hemisphere, whereas no such differences were present for the AB group. ns = not significant; *p < 0.05; **p < 0.005; ***p < 0.0005.

The circuitry of the hippocampus, with its subdivisions in the transverse axis is preserved along the longitudinal axis^44^. However, external and internal connectivity as well as neuronal morphology and gene expression differ along the septo-temporal axis of the hippocampus^16, 18, 45^. We thus analyzed whether within the dorsal part of the hippocampus size of and overlap between neuronal representations would differ along the longitudinal axis. Whereas no consistent differences were found between rostral and caudal parts of the dorsal DG (F_2, 20_ = 1.829, *p* = 0.1864; two-way ANOVA), we did find higher overall percentages of activated neurons in both groups in rostral than caudal parts of dorsal CA3 (F_2, 20_ = 14.08, *p* = 0.0002, two-way ANOVA; AA: rostral vs caudal: *p* = 0.0073; AB: rostral vs caudal: *p* = 0.0019; Bonferroni’s multiple comparisons test; Fig. 4a), suggesting that contextual representations comprise larger parts of the neuronal population at rostral than caudal parts of dorsal CA3. We also found that the similarity score after two sequential explorations was higher in rostral than caudal parts of CA3 (Fig. 4b, F2, 20 = 14.72, *p* = 0.0001, two-way ANOVA; AA: rostral vs caudal: *p* = 0.0006; AB: rostral vs caudal: *p* = 0.0181; Bonferroni’s multiple comparisons test), suggesting that neuronal representations in general have more overlap in rostral than caudal parts of dorsal CA3 possibly suggesting more stable contextual representations.

Inspired by reports on functional lateralization within the hippocampal formation^23, 24^ we finally tested whether contextual representations would differ between left and right hemispheres. Regarding the size of the contextual representations, we found that the percentage of all activated neurons in either AA or AB groups was not different between left and right hemispheres, neither in DG nor in CA3 (DG: F_1, 14_ = 0.6335, *p* = 0.4394; CA3: F_1, 14_ = 1.661, *p* = 0.2184; two-way ANOVA) (Fig. 4c). In addition, the similarity score for groups AA and AB did not differ between left and right DG (F_1, 14_ = 0.0178, *p* = 0.8957; two-way ANOVA). In contrast, CA3 representations had a significantly higher similarity score on the right than on the left side in the AA but not the AB group (Fig. 4d; F_1, 14_ = 23.71, *p* = 0.0002; two-way ANOVA; AA: right vs left: *p* = 0.0004; AB: right vs left: *p* = 0.2100; Bonferroni’s multiple comparisons test), indicating that neuronal representations of sequential explorations of the same environment have more overlap in the right than the left CA3 region.

### Discussion

In this study we have taken advantage of the time-dependent localization of zif268 mRNA to investigate features of neuronal representations simultaneously in different subregions of the DG and CA3 in response to novel or familiar contextual exposures. To our knowledge this is the first study to use the same IEG for direct comparison of DG and CA3 subregions. Much of the data we collected with this approach confirms previous results. However, we were also able to gain some novel insights into region-specific characteristics of hippocampal representations.

In previous studies, mainly catFISH for Arc/Arg3.1 or a combination of Arc/Arg3.1 and Homer1a has been used for the investigation of CA3 or CA1^2, 22, 31, 32^. For the DG zif268 has been used in some studies^36, 41, 46^. However, catFISH for the same IEG has not been used for simultaneous investigation of DG and CA3^47^. Our data suggests that double fluorescent in situ with intronic and exonic probes against zif268 reliably discriminates neuronal representations separated in time by about 30 min in both DG and CA3.

It has previously been reported that CA3 may display higher numbers of activated neurons than DG in response to environmental exposures^2, 31, 37^. In this study direct comparison showed the percentage of activated neurons to be between three- and four-fold higher in CA3 than in DG. Such differences in activation ratios would be consistent with basic concepts of pattern separation and completion in DG and CA3 respectively^48^. In addition, we found the size of activated ensembles in DG and CA3 to be largely independent of the exact context they represented, suggesting that the size of the neuronal representation may mainly reflect the size of the explored environment^31, 35^. Subregion-specific comparison of ensemble dimensions showed no consistent differences between CA3 subregions but revealed a significantly larger ratio of activated neurons in the spDG than in the ipDG across all exposures^41^.

When we assessed how well neuronal representations in subregions of DG and CA3 were able to disambiguate environments we found that all subregions displayed significantly more overlap between representations after repeated exposure to the same environment than after exposure to two different environments, suggesting similar abilities to differentiate between environments A and B. This was also true for the ipDG, which was not previously known to participate in the differentiation of environments.

Unfortunately, we here investigated only representations evoked by two types of environments with modest dissimilarity, which precludes any detailed analysis of pattern separation and pattern completion capabilities within DG and CA3 subregions. However, the fact that CA3 subregions similar to the results of Marrone et al.^37^ all showed significantly lower overlap after AB exposures than after AA exposures is well compatible with the proposed ability of CA3 to switch between pattern separation and completion as a function of environmental similarity^49^. If we assume that the degree of overlap between neuronal representations after AA exposures was indicative of the degree of pattern completion we can also state that pattern completion was significantly stronger in CA3a/b than in the dentate gyrus, whereas this was not the case for CA3c. These findings are congruent with the hypothesis that DG and CA3c participate in pattern separation whereas CA3a/b rather participates in pattern completion^10–12^, a division that matches the local wiring with connectivity between DG and CA3c via mossy cells and strong recurrent collaterals within distal CA3^10, 45, 50–52^. More experiments with incremental degrees of similarity between environments^49^ will be needed to adequately address subregional differences in pattern separation and pattern completion at the IEG level. Another shortcoming of our study is the low statistical power with only three to five mice per group. This has to be kept in mind for statements on non-significant results. Finally, our analysis is limited to male mice. Subsequent studies will thus have to include more subjects and will have to address potential gender differences in hippocampal representations.

Beyond sub-regional analysis along the transverse axis, we also investigated features of neuronal representations along the longitudinal axis of the dorsal hippocampus to reveal that activated neuronal ensembles comprised significantly larger portions of neurons in rostral than caudal parts of CA3. Differences in ensemble sizes have previously been shown between dorsal and ventral CA1 and CA3^53, 54^ (but see^22, 55^), but not within the dorsal hippocampus. Beyond this we showed that between these larger ensembles also the overlap after sequential exposure to either the familiar or a new environment was much higher in rostral than caudal parts of dorsal CA3. We thus propose that within dorsal CA3 contextual representations may be more stable in rostral than caudal parts. Although differences in overlap of neuronal representations have been reported for dorsal and ventral CA1 regions during odour recognition and with different cognitive strategies^22, 54^, to our knowledge this is the first report on differences in representation overlap along the longitudinal axis of CA3.

Finally, when we compared representations between hemispheres, we found that although ensemble sizes were not different, overlap of neuronal representations was stronger in the right than the left CA3 for the AA but not the AB sequence. These data suggest a functional lateralisation for CA3. Using AA overlap as a proxy, we hypothesise that mechanisms of pattern completion maybe more developed in the right than the left CA3. Interestingly, although hippocampal lateralization has been studied in humans for some time^56–59^, not many studies have investigated differences between left and right hippocampus in rodents. Indeed, previous catFISH-based analyses have often focused on only one side^12, 36, 41, 54, 60^. However, recent findings have indicated that also in rodents left and right hippocampus show functional differences^23, 61, 62^. Our data support this notion and are in line with recent calcium imaging data, which have proposed context discrimination in the left and generalization in the right DG^25^. A better understanding of interhemispheric differences in mnemonic processing may help to understand the specific deficits associated with neurological disorders such as Alzheimer’s disease with lateralized onset of pathology^63–65^.

In summary, using catFISH for the IEG zif268 we were able to reveal several new aspects regrading neuronal representations in dorsal DG and CA3. In particular we have been able to show that although ensemble sizes in the lower blade are smaller than in the upper blade the lower blade does participate in the disambiguation of environments. In addition, we found functional differences regarding context representation along the longitudinal axis of the dorsal hippocampus as well as between hemispheres.

## Materials and methods

### Mice

All experiments were performed in accordance with the German law on animal protection, reported in accordance with the ARRIVE guidelines and approved by the Animal Care and Ethics Committee of the University of Kiel (V242-7224.121-2). Nineteen male C57BL/6J wildtype mice at the age of 9 weeks were used as subjects. Mice were kept on a 12 h light/dark cycle in a temperature- and humidity-controlled room. All experiments took place during the light phase between 6 am and 6 pm. The animals were first group housed but separated into single cages eleven to thirteen days before the experiment. Mice were provided with food and water ad libitum.

### Experimental apparatus

The apparatus used was composed of two large boxes (context A and context B). Context A consisted of a 50 cm × 50 cm × 50 cm square plastic box with grey walls and with a metal floor. White cue cards with distinct symbols (a cross, a circle and two different triangles) were fixed on every wall and five objects of two different kinds (5 to 8 cm in size) were scattered over the floor. Context B was a circular plastic box with a diameter of 47 cm and 50 cm brown walls and a wooden floor. Here four identical white cue cards with a circle were fixed on the wall at 0°, 90°, 180° and 270° and five objects of four different kinds (5–8 cm in size) were placed in the box in a different spatial arrangement than those in context A.

### Behaviour

All mice were handled twice a day for 5 min (25-min interval) over six days prior to the experiment. For experiments animals were divided into five groups with three to five mice per group. The first group (home cage; hc) was composed of naive control mice which were taken directly from the home cage. Mice of the second group (5’) visited context A for 5 min. Mice of the third group (30’) explored context A for 5 min and were then placed back into their home cage located in the same room for 30 min. Mice of the fourth group (AA) explored context A for 5 min, were returned to their home cage for 25 min, located in the same room, and explored the same context again for 5 min. Mice of the fifth group (AB) visited context A for 5 min, were returned to their home cage for 25 min, located in the same room and then explored context B for 5 min. Between animals and exposures the arena was cleaned thoroughly to eliminate odour cues. At the end of the respective exposures unanaesthetized animals were killed within 30 s by decapitation.

During exposure to context A or B animals were tracked with an overhead CCTV camera. Data was recorded onto a PC and analysed using Ethovision XT software (Noldus, Wageningen; Netherlands). The arenas were virtually divided into nine equally sized zones of 278 cm^2^ (16,6 × 16,6 cm). We analysed path length as well as zone crossings for each trial.

### Histology and in situ hybridization

Brains were rapidly removed and frozen in tissue freezing medium (Leica Biosystems, Richmond, UK) on dry ice within a maximum of 2 min and stored at  − 80 °C until further processing. Coronal sections with a thickness of 20 µm were prepared using a cryostat (Leica; CM3050) collected on Polysine Adhesion Slides (Thermo Fisher Scientific, Waltham, USA) and stored at − 80 °C again. The region collected for IEG imaging extended from Bregma − 1.00 to − 2.7 mm to include the dorsal DG and hippocampus (Paxinos, Watson). Brain sections were evenly distributed over 15 slides per mouse to accommodate a similar range of rostrocaudal planes on each slide.

For the detection of the full-length and intronic mRNA of the immediate-early gene (IEG) zif268 double-label fluorescence in situ hybridization was used following previously published protocols with several alterations^2^. Probes were synthetized using fluorescein RNA labelling mix (Roche Diagnostics, Mannheim; Germany) for full-length zif268 and dioxygenin (DIG) labelling mix (Roche Diagnostics, Mannheim, Germany) for the intronic zif268 probe. Both were detected with peroxidase-conjugated anti-fluorescein or anti-dioxygenin antibodies. The peroxidase activity for full-length zif268 was detected with Cy3-tyramide conjugates, that for intronic zif268 with FITC-tyramide conjugates. Fluorescein- and Cy3-tyramide conjugates were synthetized as described previously^66, 67^. After labelling, peroxidase activity was blocked by quenching with 100 mM glycine–HCl (pH 2.0) solution containing 0.1% Tween. Subsequent steps were performed as in Webster et al. (2020). Finally, sections were counterstained with 4´,6-diamidino-2-phenylindole (DAPI) (Sigma-Aldrich, Munich, Germany).

The intronic zif268 RNA probe consists of the first 675 bp of the second intron of the NM_007913.5 transcript. DNA templates for the intronic zif268 RNA probe transcription were PCR-amplified from C57Bl6 mouse genomic DNA using the following primers: GCGAACAACCCTATGAGCAC and GCAGGAAAGGGAACAGAGAG. The amplified DNA fragment was cloned into the pBSK backbone and transcribed with T3 RNA polymerase using DIG RNA Labeling Mix according to the manufacturer protocol (all from Roche Diagnostics, Mannheim, Germany).

The DNA coding for the full length (3072bp) zif268 probe (EST clone NM_007913) in pCMV SPORT6 backbone was a gift from Hirohide Takebayashi^68^. The plasmid was linearized with EcoRI and transcribed with the T7 RNA polymerase using Fluorescein RNA Labeling Mix according to the manufacturer protocol (all from Roche Diagnostics, Mannheim, Germany).

### Image acquisition and analysis

Images were acquired on a Zeiss Axio Imager 2 with ApoTome.2 at 20x. To reduce variability, exposure times for the different wavelengths, ApoTome calibration and number of optical z-stacks (stacks: 6; optical thickness: 1.25 µm; stack thickness: 6.25 µm) remained constant for all slides. The number of tiles assembled to the final image varied from slide to slide according to the size of the dentate gyrus and CA3 at different Bregma levels. After acquisition stacks were combined to a maximum intensity projection (MIP) using ZEN 2 software (Carl Zeiss Microscopy GmbH; Oberkochen; Germany). We confirmed faithful detection of compartmental signals in MIPs by comparing signals detected in MIPs and reconstructions of their full z-stacks. In four sections of the DG the number of signals did not differ significantly (*p* > 0.05). Accordingly, MIPs were used for further analysis.

For each group of mice three to six sections per mouse, each containing all examined regions (spDG, ipDG, CA3c, CA3b, CA3a), were imaged at approximately the same Bregma levels and used for counting of cells as well as of nuclear and cytoplasmatic signals. One image from the home cage group in which both nuclear and cytoplasmic signals were clearly detectable was chosen as reference image. Acquisition parameters of this reference image were used for all subsequent imaging. Due to weaker cytoplasmic signals in CA3 compared to dentate gyrus, we used a lower threshold for CA3 than for dentate gyrus imaging in all groups. Signals were quantified manually using Fiji (ImageJ, National Institute of Health, USA) by an investigator blinded to group affiliations. Signals of the intrinsic probe, which did not overlap with DAPI and those, which were not also detectable with the full-length probe have not been considered. Similarly, signals from the full-length probe, which exclusively overlapped with DAPI have not been considered. We distinguished four categories of cells: 1.) cells that only displayed nuclear signals, 2.) cells that only displayed cytoplasmic signals, 3.) cells, which showed both nuclear and cytoplasmatic signals, 4.) cells that showed no signals. Sub-regions of dentate gyrus (spDG and ipDG) and CA3 (CA3a, b, c) were delineated according to previous examples^40, 44, 69^.

The total number of cells was quantified using DAPI staining. For this purpose, each subregion (spDG; ipDG; CA3c; CA3b; CA3a) was evaluated separately using CellProfiler software (Broad Institute, Massachusetts, USA), which automatically separated the nuclei from each other. The accuracy of software-based automatic quantification was validated in 5 samples consisting of spDG and CA3 each by manual quantification (automatic: 338.9 ± 61.13 SEM; manual: 335.3 ± 62.3 SEM; *p* > 0.05; Wilcoxon matched-pairs signed rank test).

### Statistical analysis

Statistical analysis was done using GraphPad Prism version 7.0d. All results were tested for normal distribution using the Shapiro–Wilk test. Control groups (hc; 5'; 30') were compared using one-way ANOVA with Bonferroni test as post-hoc test or using the Kruskal–Wallis test if the data was not normally distributed. For comparison of cell activity and similarity score between groups AA and AB a two-way ANOVA was used with Bonferroni’s multiple comparisons test as post-hoc test. Within groups the similarity score was contrasted between subregions of DG and CA3 using one-way ANOVA with Bonferroni’s multiple comparisons test or Kruskal–Wallis test if the data was not normally distributed. For the comparison of signals along the rostro-caudal axis, slices were divided into rostral, middle, and caudal slices based on their bregma-levels (rostral: Bregma − 1.2 to − 1.5 mm; middle: Bregma − 1.5 to − 1.8 mm; caudal: Bregma: − 1.8 to − 2.1 mm) and examined using a two-way ANOVA with Bonferroni’s multiple comparisons test as post-hoc test. The same tests were also performed for the comparison between right and left hemisphere. The significance level was set at 0.05 for all tests used. One mouse from the AA group was excluded from further analysis due to low quality of the in-situ hybridization with the exonic probe resulting in low signal to noise ratios for cytoplasmatic signals.

The degree of overlap between neuronal populations activated by two sequential contextual exposures was quantified using the similarity score according to previous publications^27, 30^. A similarity score of 1 corresponds to a complete reactivation of all previously activated cells. A score of 0 corresponds to the activation of two independent cell populations.

Unless stated otherwise, all data are shown as mean ± SEM. In figures bars represent means, bars with error bars refer to means ± SEMs. Circles and dots represent individual data points.

## Data Availability

The data from this study are available from the corresponding author upon reasonable request.
